# Proliferative multifocal leukoplakia better name that proliferative verrucous leukoplakia

**DOI:** 10.1186/1477-7819-9-122

**Published:** 2011-10-10

**Authors:** Jose M Aguirre-Urizar

**Affiliations:** 1 Oral Medicine Unit, Oral and Maxillofacial Pathology Unit, Dental Clinic Service. Department of Stomatology, University of the Basque Country/EHU, Barrio Sarriena s/n, Leioa. 48940, Vizcaya, Spain

## Abstract

In this letter I propose the name "Proliferative Multifocal Leukoplakia" with the goal of reducing under-diagnosis of this disease, improve the early diagnosis, try to make an early therapy and control, and prevent its malignant transformation.

## Correspondence

Dear Editor,

It has recently been published in World Journal of Surgical Oncology a progressive case report of the still enigmatic process "Proliferative Verrucous Leukoplakia" (PVL) [1].

Since the first description by Hansen et al. [2] of these oral premalignant disorder, the "verrucous appearance" seemed to be a key semiological data for the diagnosis of this particular disease that still has many unresolved issues in relation to its pathogenesis, diagnosis and treatment [3,4].

Several series of cases of PVL proving in an unchallengeable manner their high premalignant capacity have been published [5-7]. Furthermore, these series of cases have also shown that the premalignat capacity of PVL is even higher than the one observed in "common leukoplakias". Therefore, the early diagnosis of this pathology plays a crucial role when trying to prevent a malignant transformation in PVL or at least when trying to prevent de development of extensive carcinomas. Typically many of the cases described in the literature have been diagnosed in a retrospective fashion, including the initial study of Hansen et al. [2].

In a recently published review article [8], major and minor diagnostic criteria have been proposed for diagnosing PVL, including "the presence of a verrucous area" between the major diagnostic criteria.

To our knowledge, we believe that this criteria should not be consider a major criteria in this disease, since in many cases the initial lesions are not warty, and when they finally have a verrucous appearance, they histologically correspond to verrucous carcinomas [4,7,8]. Therefore, if you consider the verrucous appearance as a major criterion, the diagnosis of this disease may happen to be delayed.

For my experience, I believe that the most important diagnostic criteria for this type of leukoplakia, which should be recognized early, are its "proliferate" and "multifocal" nature. The proliferative nature of this oral pathology would conditioned by the following elements: the existence of multiple leukoplakias (more than 2 locations), registering the clinical growth of the lesions and the relapse of previously treated areas in a suitable way. Logically, the performance and assessment of representative biopsies is essential to establish the existence of epithelial dysplasia or a carcinoma.

These considerations are in agreement with the views of other authors such as Saito et al. [9] and Hamadah et al. [10], who pointed out that "widespread multiple oral lesions" were the ones with a greater malignant potential when compared with the ones which were localized and unique.

Therefore, I propose the name "Proliferative Multifocal Leukoplakia" for this disease with the goal of reducing under-diagnosis, improve the early diagnosis, try to make an early therapy and control, and prevent its malignant transformation.

## Competing interests

The author declares that they have no competing interests.

## References

[B1] Ge L Wu Y Wu LY Zhang L Xie B Zeng X Lin M Zhou HM Case report of rapidly progressive proliferative verrucous leukoplakia and a proposal for aetiology in mainland China World J Surg Oncol 2011 9 26 10.1186/1477-7819-9-26 21352571 PMC3056810

[B2] Hansen LS Olson JA Silverman S Jr Proliferative verruocus leukoplakia. A long-term study of thirty patients Oral Surg Oral Med Oral Pathol 1985 60 285 98 10.1016/0030-4220(85)90313-5 3862042

[B3] van der Waal I Reichart PA Oral proliferative verrucous leukoplakia revisited Oral Oncol 2008 44 719 21 10.1016/j.oraloncology.2007.09.010 18061520

[B4] Bagan J Scully C Jimenez Y Martorell M Proliferative verrucous leukoplakia: a concise update Oral Dis 2010 16 328 32 10.1111/j.1601-0825.2009.01632.x 20233330

[B5] Zakrzewska JM Lopes V Speight P Hopper C Proliferative verrucous leukoplakia: a report of ten cases Oral Surg Oral Med Oral Pathol Oral Radiol Endod 1996 82 396 401 10.1016/S1079-2104(96)80303-9 8899776

[B6] Silverman S Jr Gorsky M Proliferative verrucous leukoplakia: a follow-up study of 54 cases Oral Surg Oral Med Oral Pathol Oral Radiol Endod 1997 84 154 7 10.1016/S1079-2104(97)90062-7 9269017

[B7] Bagan JV Jimenez Y Sanchis JM Poveda R Milian MA Murillo J Scully C Proliferative verrucous leukoplakia: high incidence of gingival squamous cell carcinoma J Oral Pathol Med 2003 32 379 82 10.1034/j.1600-0714.2003.00167.x 12846783

[B8] Cerero-Lapiedra R Baladé-Martínez D Moreno-López LA Esparza-Gómez G Bagán JV Proliferative verrucous leukoplakia: a proposal for diagnostic criteria Med Oral Pat Oral Cir Bucal 2010 15 839 45 20173704

[B9] Saito T Sugiura C Hirai A Notani K Totsuka Y Shindoh M Kohgo T Fukuda H High malignant transformation rate of widespread multiple oral leukoplakias Oral Dis 1999 5 15 9 10218036 10.1111/j.1601-0825.1999.tb00058.x

[B10] Hamadah O Goodson ML Thomson PJ Clinicopathological behaviour of multiple oral dysplastic lesions compared with that of single lesions Br J Oral Maxillofac Surg 2010 48 503 6 10.1016/j.bjoms.2009.08.027 19800717

